# Knowledge, attitudes, and behavioural intention towards HIV pre-exposure prophylaxis among health science students in South Africa: cross-sectional study

**DOI:** 10.3389/fpubh.2026.1858282

**Published:** 2026-06-11

**Authors:** Tshepo A. Ntho, Molatelo M. Rasweswe, Ledile E. Manamela, Mxolisi W. Ngwenya

**Affiliations:** Department of Nursing Science, Faculty of Health Sciences, University of Limpopo, Polokwane, Limpopo, South Africa

**Keywords:** health science students, HIV pre-exposure prophylaxis, knowledge, attitudes, behavioural intention, South Africa, nursing, pharmacy

## Abstract

**Background:**

Advanced HIV disease remains a significant public health challenge in Sub-Saharan Africa, with inadequate uptake of biomedical prevention strategies such as HIV Pre-Exposure Prophylaxis (PrEP) contributing to persistent new infections and disease progression.

**Objective:**

This study aimed to assess the knowledge, attitudes, and behavioural intention towards HIV PrEP among health science students at a selected Higher Education Institution (HEI) in Limpopo Province, South Africa.

**Methods:**

A cross-sectional descriptive study was conducted among 350 health science students. Data were collected using a self-administered structured questionnaire and analysed using descriptive statistics and chi-square analyses in SPSS version 28.0, with statistical significance set at *p* < 0.05.

**Results:**

Most health science students demonstrated good knowledge (88.6%) and positive attitudes (79.7%) towards PrEP, with high behavioural intention (80.3%). Social media was the most frequently cited source of PrEP awareness. Despite the overall high knowledge levels, important gaps were identified regarding PrEP eligibility and dosing frequency. In addition, nearly half of the participants expressed risk compensation concerns related to PrEP use. Notably, age (*p* = 0.036) and year of study (*p* = 0.026) were significantly associated with both knowledge and attitudes, with older and more academically advanced students demonstrating comparatively stronger performance across both domains, while gender, relationship status, and study programme were not significantly associated with any outcome measure.

**Conclusion:**

Despite predominantly good knowledge and positive attitudes, persistent knowledge gaps and misconceptions about risk compensation concerns underscore the need for curriculum-integrated PrEP education and strengthened campus-based HIV prevention services, particularly in the context of the UNAIDS 95-95-95 targets, the Sustainable Development Goals, and the African Union Agenda 2063.

## Introduction

Advanced HIV Disease (AHD), defined by the World Health Organization (WHO) as a CD4 cell count below 200 cells/mm^3^ or the presence of a WHO clinical stage 3 or 4 condition in adults and adolescents, remains a major public health challenge across Sub-Saharan Africa (1). Despite remarkable global progress in expanding access to antiretroviral therapy (ART), AHD continues to drive unacceptably high mortality rates, largely due to opportunistic infections such as tuberculosis (TB), cryptococcal meningitis, and *Pneumocystis jirovecii* pneumonia (2). South Africa bears a disproportionate burden, with approximately 7.5 million people living with HIV and, in 2021 alone, an estimated 210,000 new infections and 51,000 AIDS-related deaths, confirming its status as the epicentre of the global epidemic (3). A defining feature of AHD is late diagnosis and delayed ART initiation, resulting in high viral loads, rapid immune deterioration, and increased HIV transmission risk (4). Patients presenting with advanced disease place considerable strain on healthcare systems due to the need for intensive management of multiple opportunistic infections (2). They often require intensive clinical management for multiple, concurrent opportunistic infections. Addressing AHD therefore requires urgent upstream interventions focused on preventing new infections, promoting early diagnosis, and ensuring timely linkage to care before immune compromise occurs. Importantly, primary prevention strategies, including biomedical interventions such as HIV PrEP, are central rather than complementary to reducing AHD (5).

Globally, the Joint United Nations Programme on HIV/AIDS has established the 95-95-95 targets to end the HIV epidemic by 2030 (6). These targets aim for 95% of people living with HIV to know tr status, 95% of diagnosed individuals to receive sustained ART, and 95% of those on treatment to achieve viral suppression. Achieving these goals requires not only treatment optimization but also a strong emphasis on preventing new infections. Reducing HIV incidence is critical to preventing progression to AHD and associated morbidity and mortality (5). PrEP has emerged as one of the most effective biomedical HIV prevention strategies. Clinical trials demonstrate that daily oral tenofovir disoproxil fumarate/emtricitabine significantly reduces HIV acquisition among high-risk populations (7). Following the United States Food and Drug Administration approval of Truvada for PrEP in 2012, the Centres for Disease Control and Prevention confirmed that PrEP reduces the risk of sexually acquired HIV by up to 99% (8). In 2015, the WHO recommended PrEP as part of a combination HIV prevention for individuals at substantial risk, including young people and key populations (9). South Africa subsequently integrated PrEP into national HIV guidelines, with implementation in selected primary healthcare facilities (10).

Despite this efficacy, PrEP uptake remains low in practice. Evidence consistently highlights a gap between awareness and utilization (5, 11–13). Barriers include stigma, misconceptions about side effects, low perceived HIV risk, adherence burden, mental health challenges, and structural constraints such as transport and cost (12, 14–16). In South Africa, PrEP awareness among young people remains low, with less than half reporting knowledge of it (13, 17). This is particularly concerning as young adults aged 18–32 years account for a substantial proportion of new HIV infections nationally (18). Delayed or absent PrEP uptake in this group increases ongoing transmission and future progression to AHD. Beyond individual-level barriers, structural determinants such as poverty, gender inequality, low health literacy, and weak healthcare systems further limit PrEP scale-up (13, 19–21). In Limpopo Province, these challenges are intensified by rural–urban disparities, limited youth-friendly services, and barriers to timely HIV testing and linkage to care (13, 22, 23). These contextual realities reflect broader determinants of AHD in SSA, where delayed diagnosis and treatment initiation continue to drive disease progression (2, 4, 11). Strengthening upstream prevention is therefore essential to reduce both new infections and AHD burden.

HEIs provide a strategic platform for HIV prevention interventions. University students, particularly those experiencing increased independence and experimentation, may be at heightened risk of HIV acquisition and thus represent a key prevention population (13, 24). Students in health-related disciplines such as Nursing and Pharmacy are especially important, as they are both potential PrEP users and future healthcare providers whose knowledge, attitudes, and behavioural intentions influence patient education, clinical decision-making, and stigma reductio (25, 26). Strengthening tr PrEP literacy may therefore improve both personal protection and broader public health outcomes, including earlier prevention of HIV infection and reduced AHD burden. However, evidence indicates persistent gaps in PrEP knowledge among health science students globally. In the United States of America, a study found low awareness of injectable PrEP, limited knowledge, and poor familiarity with prescribing guidelines (27). Similarly, in Spain, nursing students demonstrated low PrEP knowledge and neutral attitudes towards its use (28). Likewise, Sub-Saharan Africa continues to report low PrEP uptake despite proven efficacy, partly due to inadequate knowledge (5, 29). In South Africa, although PrEP has been incorporated into national HIV prevention guidelines since 2016, evidence on PrEP knowledge, attitudes, and behavioural intentions among tertiary students remains limited, particularly in Limpopo Province. Given the high HIV burden, structural healthcare challenges, and limited access to youth-friendly services in this setting, there is a clear need for empirical evidence to inform targeted interventions (22, 23). This study, therefore, aims to assess PrEP knowledge, attitudes, and behavioural intention among Nursing and Pharmacy students in a selected HEI in Limpopo Province. The findings are expected to inform curriculum strengthening, campus-based HIV prevention programmes, and institutional health services, ultimately contributing to improved early prevention strategies and reduced AHD-related morbidity and mortality.

## Methods

### Research design and setting

A cross-sectional descriptive study design was employed, which is appropriate for examining the prevalence of health-related knowledge, attitudes, and behavioural intention at a single point in time. The study was conducted at a selected HEI located in the Capricorn District, approximately 32 kilometres east of Polokwane, the provincial capital of Limpopo Province. The institution is among the historically disadvantaged HEIs in South Africa, serving a predominantly rural student population. The HEI comprises four faculties, including the Faculty of Health Sciences, which is subdivided into the School of Medicine and the School of Health Care Sciences (SHCS). The SHCS comprises four departments, namely Pharmacy, Nursing Science, Human Nutrition and Dietetics, and Optometry. Notably, Nursing Science and Pharmacy were purposively selected as the focus of this study due to their direct curricular engagement with HIV prevention through health education and pharmacological interventions. Both the Bachelor of Nursing Science and the Bachelor of Pharmacy are four-year professional degree programs accredited by the South African Nursing Council under Regulation No. 174 of 2013 and the South African Pharmacy Council (SAPC) under the Pharmacy Act (Act 53 of 1974), respectively, equipping graduates as future frontline healthcare providers with a critical role in HIV prevention and PrEP promotion.

### Population and sampling

The target population comprised all undergraduate students enrolled in the Departments of Nursing Science and Pharmacy within the School of Health Care Sciences, totalling 623 students (Nursing Science = 333; Pharmacy = 290). Eligible respondents were undergraduate students registered in these departments for the 2024 academic year across all levels of study who provided informed consent to participate. A stratified convenience sampling approach with proportionate allocation was employed in this study. The population was first stratified according to academic department to ensure adequate representation from both groups, after which participants were recruited through voluntary participation within each stratum. The sampling process and participant recruitment procedure are illustrated in Supplementary Figure S1. The minimum required sample size for each stratum was determined separately using Slovin’s formula:

$$n=\frac{N}{1+N(e^{2})}$$

Where *n* represents the sample size, *N* represents the population size, and (*e*^2^) represents the margin of error. Using a 95% confidence level and a margin of error of 0.05, the minimum required sample size was calculated separately for each department. Application of the formula to the Nursing Science (*N* = 333) and Pharmacy (*N* = 290) strata yielded minimum sample sizes of 182 and 168 participants, respectively, resulting in a combined minimum sample of 350 participants. A study information flyer containing the survey link was distributed via the respective departmental WhatsApp groups to all 623 eligible undergraduate students in both departments. Participants were recruited through voluntary self-selection within each stratum, and data collection continued until the minimum required sample size for each department was achieved. A total of 182 completed questionnaires were obtained from Nursing Science students and 168 from Pharmacy students, corresponding to response rates of 54.7 and 57.9%, respectively. All completed questionnaires were included in the final analysis. Given the voluntary nature of participation and the online recruitment approach, the possibility of self-selection bias cannot be excluded, and the findings may therefore have limited generalisability beyond the study population.

### Data collection

Data were collected between 06 September and 23 October 2024 using a structured, self-administered online questionnaire designed to assess undergraduate students’ knowledge, attitudes, and behavioural intention towards HIV PrEP. The instrument comprised 22 items, including five sociodemographic questions, six knowledge-based items, seven attitude items, and four behavioural intention items assessing willingness to use PrEP under different scenarios. The instrument was developed from relevant literature and adapted to the local study context to ensure content relevance and appropriateness for Nursing and Pharmacy students (11, 17, 30–32). The questionnaire was hosted on Google Forms, and the survey link was distributed via a study information flyer circulated through the WhatsApp groups of Nursing and Pharmacy students. Interested health science students accessed the questionnaire by clicking the link provided in the flyer, which redirected them to the online survey platform. The Google Form was preceded by an informed consent page providing detailed information about the study objectives, procedures, and ethical considerations. Respondents were required to indicate their consent before proceeding with the questionnaire. Importantly, if consent was not provided, access to the survey was automatically terminated. Participation was entirely voluntary, and respondents were informed of their right to withdraw at any stage before submission. Anonymity and confidentiality were strictly maintained, as no personally identifiable information was collected.

### Study instrument

A self-administered structured questionnaire was used for data collection, organised into four sections. Notably, before the main data collection, the questionnaire was pilot tested among 35 undergraduate students from the Departments of Human Nutrition and Dietetics and Optometry, representing 10% of the calculated study sample. The pilot test assessed the clarity and appropriateness of the questionnaire items, and feedback obtained informed minor revisions to the wording and structure of selected items in consultation with the study supervisors and biostatistician. Section A collected sociodemographic data, including age, gender, relationship status, level of study, and academic programme. Section B assessed PrEP knowledge using six items covering awareness, definition, mechanism of action, eligibility, dosing frequency, and benefits. Items were presented in dichotomous and multiple-choice formats and recoded into binary variables, with correct responses scored as 1 and incorrect or unknown responses as 0, yielding a composite score ranging from 0 to 6. Scores were classified into poor knowledge (0–3) and good knowledge (4–6), consistent with approaches used in comparable HIV prevention research (33, 34). Notably, detailed distributions of responses to individual knowledge items are presented in Supplementary Table S1.

Section C assessed attitudes towards PrEP using seven items covering perceived effectiveness, risk compensation concerns, willingness to use PrEP, its importance in HIV prevention, adherence, and misconceptions related to condom use and multiple sexual partnerships. Items were measured using mixed response formats and recoded into a uniform three-point scale (1 = negative attitude, 2 = neutral, 3 = positive attitude), with reverse coding applied to negatively worded items. Total scores ranged from 7 to 21, with the midpoint (15) used as the cut-off to classify respondents into negative and positive attitude groups (28, 35). Item-level responses are presented in Supplementary Table S2. The internal consistency of the attitude scale was assessed using Cronbach’s alpha, yielding a value of 0.451. Although this value falls below the conventionally accepted threshold of 0.70, the scale was retained due to the theoretical relevance of the included items in assessing different aspects of PrEP-related attitudes among health science students. The relatively low reliability coefficient should, however, be considered when interpreting the attitude-related findings. Section D assessed behavioural intention towards PrEP using four items measuring willingness to use PrEP under different scenarios, general willingness, increased access to information, free availability, and availability without prescription. Each item was measured on a three-point scale (Yes = 3, Maybe = 2, No = 1). A composite score ranging from 4 to 12 was computed, with scores classified into low (4–8) and high (9–12) behavioural intention categories based on the scale midpoint. Item-level responses are presented in Supplementary Table S3.

### Data analysis

Data were analysed using the Statistical Package for the Social Sciences (SPSS) version 28.0. Descriptive statistics were used to summarise sociodemographic characteristics and responses to knowledge, attitude, and behavioural intention items, with categorical variables reported as frequencies and percentages. The internal consistency of the knowledge, attitude, and behavioural intention scales was assessed using Cronbach’s alpha, yielding values of 0.680, 0.451, and 0.810, respectively, indicating acceptable, low, and good reliability across the three scales. Chi-square tests of independence were conducted to examine associations between sociodemographic characteristics, including age category, gender, relationship status, year of study, and study programme, and the three outcome variables. Fisher’s Exact Test was applied where expected cell frequencies fell below five. The level of statistical significance was set at *p* < 0.05 for all analyses.

### Ethical considerations

Ethical approval for the study was granted by the Turfloop Research Ethics Committee (TREC/441/2024: UG), and institutional permission was obtained from the Director of the School of Health Care Sciences and the Head of the Department of Nursing Sciences. Participation was entirely voluntary, and all respondents provided written informed consent prior to data collection. Respondents were informed of the study’s purpose, their right to withdraw at any stage without penalty, and the absence of academic consequences for non-participation. Anonymity and confidentiality were maintained throughout; questionnaires were completed anonymously, no identifying information was collected, and data were stored securely with access restricted to the research team. The study was conducted in accordance with the ethical principles of the Declaration of Helsinki, encompassing respect for persons, beneficence, and justice.

## Results

### Sociodemographic profile of health science students

A total of 350 students participated in the study, with 49.4% aged 20 years and below and 50.6% aged 21 years and above. The sample was predominantly female (74.6%), with male respondents accounting for 25.4%. The majority of respondents were single (95.7%), with only 4.3% reporting being in a romantic relationship. With regard to level of study, the largest proportion of respondents were in their fourth year (29.1%), followed by third year (26.3%), first-year (24.0%), and second-year students (20.6%). Participation was nearly equally distributed between the two programmes, with Nursing students comprising 51.7% and Pharmacy students 48.3% of the sample (see Table 1).

**Table 1:** Sociodemographic profile of nursing and pharmacy students (*n* = 350).

Variable	Category	*n* (%)
Age (Years)	≤20 years	173 (49.4)
	≥21 years	177 (50.6)
Gender	Male	89 (25.4)
	Female	261 (74.6)
Relationship status	Single	335 (95.7)
	Relationship	15 (4.3)
Level of study	1st level	84 (24.0)
	2nd level	72 (20.6)
	3rd level	92 (26.3)
	4th level	102 (29.1)
Programme	Nursing	181 (51.7)
	Pharmacy	169 (48.3)

Participant characteristics (*n* = 350); *n* = number of respondents.

### First source of PrEP awareness among health science students

**Figure 1:**
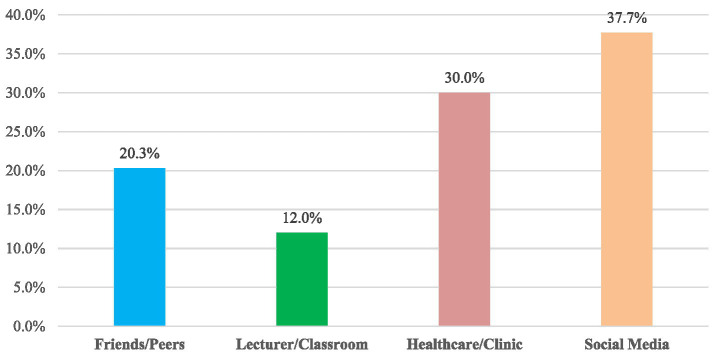
Source of PrEP awareness among health science students.

As depicted in Figure 1, social media was the most frequently cited primary source through which respondents first heard about PrEP (37.7%), followed by healthcare facilities or clinics (30.0%), friends or peers (20.3%), and lectures or classroom teaching (12.0%).

### Knowledge of HIV PrEP among health science students

**Figure 2:**
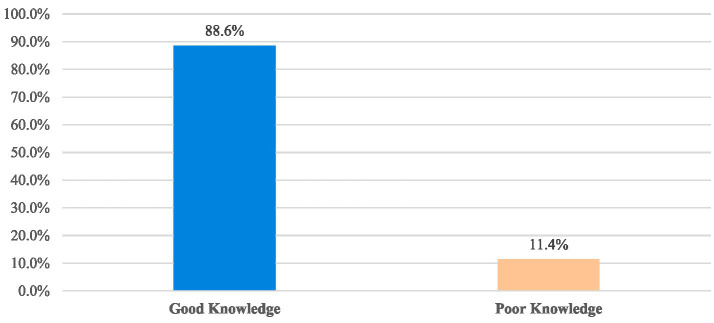
Overall, knowledge classification among health science students.

Overall, 88.6% of respondents met the study-defined threshold for good knowledge of PrEP, with a mean knowledge score of 0.89 ± 0.32 and a median of 1.00, indicating generally high levels of PrEP awareness among participants (Figure 2). However, item-level analysis revealed important gaps in specific areas of knowledge, particularly regarding PrEP eligibility and dosing frequency. The majority of respondents (90.0%) reported having heard of PrEP prior to the study, with only 10.0% indicating no prior awareness. When asked to define PrEP, most respondents correctly identified it as a medication to prevent HIV (87.7%), while smaller proportions incorrectly described it as a vaccine against HIV (6.6%), a treatment for HIV (1.1%), or indicated they did not know (4.6%). Regarding the mechanism of action, 71.4% correctly identified that PrEP works by preventing HIV from entering the body, while 12.9% indicated they did not know, 12.3% believed it boosts the immune system, and 3.4% incorrectly stated that it kills HIV in the body. With respect to eligibility, responses were more divided, with 47.4% indicating that anyone can take PrEP and 45.4% correctly identifying that PrEP is intended only for people at high risk of HIV infection, while 7.1% were unsure. Concerning dosing frequency, 68.3% correctly identified that PrEP should be taken daily. However, 26.6% indicated they did not know, and the remainder reported weekly (1.7%) or monthly (3.4%) intervals. Regarding the benefits of PrEP, the large majority (88.9%) correctly identified that PrEP reduces the risk of HIV infection, while 5.7% did not know and 4.9% believed it boosts the immune system (see Supplementary Table S2 for full item-level response distributions).

### Attitude of health science students towards HIV PrEP

**Figure 3:**
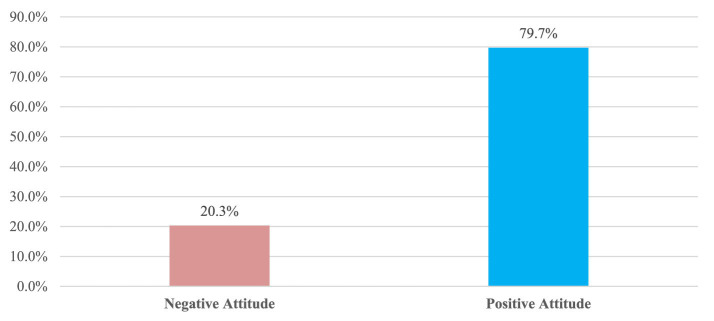
Overall attitude classification among health science students.

Regarding attitudes towards PrEP, overall 79.7% of respondents held a positive attitude, 20.3% a negative attitude, with a mean attitude score of 0.80 ± 0.40 and a median of 1.00, indicating that attitudes were predominantly positive among nursing and pharmacy students (Figure 3). The majority of respondents (76.9%) believed that PrEP is an effective way to protect oneself from HIV, while 14.9% were unsure and 8.3% did not. With respect to risk compensation concern, nearly half of respondents (48.6%) agreed that PrEP promotes risky sexual behaviour, 32.3% disagreed, and 19.1% were uncertain, reflecting a notable misconception among a substantial proportion of the sample. The majority of respondents (91.4%) indicated they would consider taking PrEP if they were at high risk of HIV infection, with only 2.6% indicating they would not and 6.0% remaining undecided. Concerning the role of PrEP in HIV prevention, most respondents either strongly agreed (49.1%) or agreed (36.9%) that PrEP is an important HIV prevention strategy, with only a small minority expressing neutrality (12.9%) or disagreement (1.2%). Similarly, the overwhelming majority strongly agreed (72.6%) or agreed (22.3%) that it is necessary to follow PrEP instructions strictly, with only 4.6% uncertain and 0.6% disagreeing. With regard to misconceptions about sexual behaviour while on PrEP, the majority disagreed (31.4%) or strongly disagreed (42.0%) that having multiple sexual partners is acceptable when using PrEP, while 16.6% were uncertain, and a minority agreed (5.1%) or strongly agreed (4.9%). Likewise, most respondents rejected the notion that condom use is unnecessary when taking PrEP, with 59.1% strongly disagreeing and 25.1% disagreeing. In comparison, 9.4% were uncertain, and a small proportion agreed (2.9%) or strongly agreed (3.4%) (see Supplementary Table S3 for full item-level response distributions).

With respect to behavioural intention towards PrEP, the majority of respondents (74.0%) indicated they would use PrEP as an HIV prevention method, while 10.9% would not and 15.1% remained uncertain. Willingness to use PrEP was notably influenced by contextual and structural factors. The majority indicated greater willingness if PrEP were available free of charge (79.1%) and if they had access to more information (78.9%), with only 4.9 and 5.4% disagreeing, respectively. Responses were, however, more divided regarding prescription accessibility, where approximately half (50.9%) indicated willingness to use PrEP if available without a prescription, while 32.9% disagreed and 16.3% remained uncertain. These findings suggest that while behavioural intention towards PrEP is generally positive, it is largely contingent upon the removal of structural barriers, particularly cost, information access, and prescription requirements.

### Behavioural intention among health science students towards HIV PrEP

**Figure 4:**
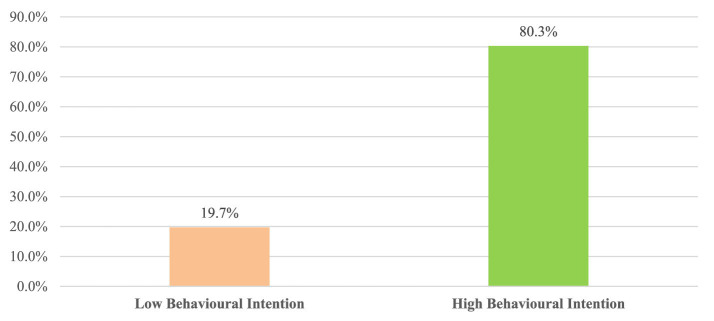
Behavioural intention among health science students towards HIV PrEP.

Overall, the majority of respondents demonstrated high behavioural intention towards PrEP use (80.3%), while 19.7% demonstrated low behavioural intention, with a mean behavioural intention score of 1.80 ± 0.40 and a median of 2.00, indicating that despite the noted contextual barriers, particularly around cost and accessibility, behavioural intention towards PrEP use was predominantly high across the sample (Figure 4). With respect to behavioural intention towards PrEP, the majority of respondents (74.0%) indicated they would use PrEP as an HIV prevention method, while 15.1% were unsure and 10.9% would not. The willingness to use PrEP was notably influenced by contextual factors: 78.9% reported they would be more willing to use PrEP if they had access to more information, 15.7% were unsure, and 5.4% disagreed. Similarly, 79.1% indicated they would be more willing to use PrEP if it were available free of charge, with 16.0% uncertain and 4.9% disagreeing. With regard to accessibility, responses were more divided, with 50.9% indicating they would be more willing to use PrEP if it were available without a prescription, 32.9% disagreeing, and 16.3% remaining uncertain (see Supplementary Table S4 for full item-level response distributions).

### Association between sociodemographic characteristics and PrEP knowledge, attitudes, and behavioural intention

Table 2 presents Chi-square analyses which were conducted to examine associations between sociodemographic characteristics and PrEP knowledge, attitudes, and behavioural intentions. With respect to age, statistically significant associations were found between age category and both knowledge (*χ*^2^(1) = 4.381, *p* = 0.036) and attitude (*χ*^2^(1) = 4.418, p = 0.036), with older students (≥21 years) demonstrating higher proportions of good knowledge (92.1%) and positive attitudes (84.2%) towards PrEP compared to tr younger counterparts (≤20 years) who showed good knowledge of 85.0% and positive attitudes of 75.1%. No statistically significant association was found between age category and behavioural intention (*χ*^2^(1) = 3.432, *p* = 0.064). With respect to gender, no statistically significant associations were found between gender and knowledge (*χ*^2^(1) = 0.498, *p* = 0.481), attitude (*χ*^2^(1) = 0.083, *p* = 0.773), or behavioural intention (*χ*^2^(1) = 1.889, *p* = 0.169) towards PrEP. Similarly, relationship status was not significantly associated with knowledge (*χ*^2^(1) = 2.022, *p* = 0.155), attitude (*χ*^2^(1) = 0.468, *p* = 0.494), or behavioural intention (*χ*^2^(1) = 1.686, *p* = 0.194). Study programme was likewise not significantly associated with knowledge (*χ*^2^(1) = 1.536, *p* = 0.215), attitude (*χ*^2^(1) = 0.006, *p* = 0.940), or behavioural intention (*χ*^2^(1) = 0.795, *p* = 0.372). Year of study demonstrated statistically significant associations with both knowledge (*χ*^2^(3) = 9.227, *p* = 0.026) and attitude (*χ*^2^(3) = 10.881, *p* = 0.012), with higher proportions of good knowledge and positive attitudes observed among students in advanced years of study, particularly fourth-year students (93.1 and 89.2% respectively), compared to first-year students (79.8 and 70.2% respectively). No statistically significant association was observed between year of study and behavioural intention (*χ*^2^(3) = 3.449, *p* = 0.327).

**Table 2:** Association between sociodemographic characteristics and PrEP knowledge, attitude, and behavioural intention among.

Variable	Category	Good knowledge *n* (%)	*χ* ^2^	*p*	Positive attitude *n* (%)	*χ* ^2^	*p*	High BI *n* (%)	*χ^2^*	*p*
Age	≤20 years	147 (85.0)	4.381	0.036*	130 (75.1)	4.418	0.036*	132 (76.3)	3.432	0.064
	≥21 years	163 (92.1)			149 (84.2)			149 (84.2)		
Gender	Male	77 (86.5)	0.498	0.481	70 (78.7)	0.083	0.773	67 (75.3)	1.889	0.169
	Female	233 (89.3)			209 (80.1)			214 (82.0)		
Relationship status	Single	295 (88.1)	2.022	0.155	266 (79.4)	0.468	0.494	267 (79.7)	1.686	0.194
	In Relationship	15 (100.0)			13 (86.7)			14 (93.3)		
Level of study	1st Year	67 (79.8)	9.227	0.026*	59 (70.2)	10.881	0.012*	70 (83.3)	3.449	0.327
	2nd Year	64 (88.9)			55 (76.4)			56 (77.8)		
	3rd Year	84 (91.3)			74 (80.4)			69 (75.0)		
	4th Year	95 (93.1)			91 (89.2)			86 (84.3)		
Study programme	Nursing	164 (90.6)	1.536	0.215	144 (79.6)	0.006	0.940	142 (78.5)	0.795	0.372
	Pharmacy	146 (86.4)			135 (79.9)			139 (82.2)		

BI, Behavioural Intention; *n*, number of respondents; *χ*^2^, Pearson Chi-Square statistic; Chi-square tests were used for all variables. Fisher’s Exact Test was applied where expected cell counts were below 5. *p < 0.05 (statistically significant).

## Discussion

This study assessed the knowledge, attitudes, and behavioural intention towards HIV PrEP among health science students at a selected HEI in Limpopo Province, South Africa. The high prevalence of PrEP awareness and good overall knowledge classification observed in this study is encouraging. It provides a solid foundation for further integration of emerging prevention modalities such as the recently introduced long-acting injectable PrEP into health science curricula (26). These findings are broadly consistent with evidence from high-income settings, where PrEP literacy among health science students has generally been reported as favourable, albeit with notable disciplinary variation. Evidence from the United States indicates that although PrEP awareness and knowledge may vary across disciplines, they are generally favourable among health science students, with pharmacy students often demonstrating comparatively higher knowledge level (30). This disciplinary variation is not surprising, given that health science curricula differ substantially in their coverage of HIV prevention and biomedical interventions, which may partly explain why awareness and knowledge levels fluctuate across professional training programmes. In contrast, considerably lower levels of PrEP knowledge have been reported in other contexts, including among nursing students in Spain, where limited knowledge and generally neutral attitudes towards PrEP were documented (28). These findings suggest that even within healthcare training environments, PrEP literacy cannot be assumed. Within the South African context, evidence similarly reflects suboptimal PrEP awareness among young people, while findings from Tanzania paint an even starker picture, with only a small minority of young people reporting awareness of PrEP or endorsing its use (17, 36). The comparatively higher awareness and knowledge levels observed in the present study may therefore reflect the relatively the concentrated HIV prevention messaging directed at health science students. On the other hand, the present findings are comparable to studies conducted among healthcare workers in South Africa, where high levels of PrEP awareness have been reported among registered nurses (35). Similarly, evidence from Namibia indicates that nursing students demonstrate comparably high levels of PrEP awareness (32, 37). These findings suggest that increased exposure to healthcare training and HIV-related services may enhance PrEP literacy. Particularly, these reinforce the notion that health science training environments across the Southern African region serve as a consistent and meaningful facilitator of PrEP awareness and knowledge. Importantly, the observed variation in PrEP knowledge, and awareness across studies may also be partly explained by the absence of universally standardized measurement tools. Global health agencies such as WHO and UNAIDS recommend harmonized indicators for HIV prevention to improve comparability across settings.

Despite high awareness, specific knowledge gaps were identified that warrant attention, particularly regarding daily adherence requirements and eligibility criteria. These misconceptions carry significant clinical implications given that inconsistent use and misunderstanding of who qualifies for PrEP can substantially undermine its protective efficacy. Such gaps are consistent with the broader literature, where high awareness has repeatedly been shown not to translate into accurate functional knowledge (35, 38, 39). This distinction is critical, as awareness without accurate knowledge represents a superficial engagement with PrEP that is unlikely to translate into correct use, and appropriate help-seeking. Misunderstanding eligibility criteria is particularly consequential, as it may lead to inappropriate self-exclusion among genuinely high-risk individuals or, conversely, false reassurance among those who do not meet clinical thresholds. These findings highlight a critical need for targeted, content-specific PrEP education that moves beyond general familiarity towards clinically meaningful understanding, particularly within university and healthcare settings where structured health education interventions can be systematically delivered, monitored, and evaluated.

Social media emerged as the primary source of PrEP information among health science students, surpassing healthcare facilities and formal classroom education. These findings underscore the growing influence of digital platforms in shaping how young people access and engage with health information. This aligns with evidence that social media has become increasingly influential in disseminating health-related knowledge and supporting decision-making among younger populations (40, 41). Furthermore, social media platforms have demonstrated significant reach in HIV-specific communication, offering opportunities for rapid and wide dissemination of prevention information including PrEP (42, 43). However, while social media may facilitate heightened PrEP awareness, it simultaneously raises legitimate concerns regarding the accuracy, consistency, and clinical reliability of unregulated health content, which could distort understanding or create misconceptions about PrEP. The comparatively limited role of healthcare facilities and classroom teaching signals missed opportunities for structured, evidence-based PrEP education within both clinical and academic settings. Strengthening these institutional channels remains essential, as they are uniquely positioned to deliver accurate, contextualised, and destigmatised PrEP information that supports informed and sustained engagement among young people.

The predominantly positive attitudes towards PrEP observed in this study are consistent with an emerging body of evidence suggesting a gradual attitudinal shift among young people in Southern Africa, where growing HIV prevention literacy and increased PrEP visibility appear to be fostering greater acceptability of biomedical prevention strategies (19, 44–46). This is particularly noteworthy given the historical resistance to biomedical HIV prevention tools in many sub-Saharan African settings, where cultural, religious, and gender-related factors have previously constrained PrEP acceptability among youth (11). These findings stand in notable contrast to evidence from the United States, where attitudes towards PrEP remain sub-optimal and utilisation among Black college students is disproportionately low, a disparity driven by intersecting determinants including medical mistrust, stigma, and limited access to culturally competent healthcare (47). Similarly, in Zambia, university students have demonstrated predominantly negative attitudes towards PrEP (48). These suggest that even within the African continent, attitudinal receptivity towards PrEP is not uniform and may be shaped by country-specific differences in PrEP programme maturity, health system capacity, and the reach of targeted youth-focused HIV prevention campaigns. However, the finding that nearly half of respondents endorsed the belief that PrEP promotes risky sexual behaviour represents a significant attitudinal concern that merits careful interpretation. This risk compensation concern is well documented in the PrEP literature and has consistently emerged as a barrier to both initiation and sustained use across diverse populations (16, 24, 29, 49–51). Notably, this belief appeared to coexist alongside the majority of health science students correctly rejecting the notion that condom use becomes unnecessary on PrEP. This underscores the importance of addressing risk compensation concerns explicitly within PrEP counselling and education interventions.

Behavioural intention towards PrEP was primarily high among health science students, reflecting a strong motivational foundation for PrEP engagement that is consistent with findings from comparable young populations in Uganda, Zimbabwe and South Africa (52). However, this intention was notably contingent on contextual factors, particularly cost and access to information, suggesting that positive intention alone does not guarantee uptake in the absence of enabling structural conditions. This pattern mirrors evidence from other low- and middle-income settings, where high PrEP intention has repeatedly failed to translate into actual uptake due to persistent financial and informational barriers that disproportionately affect young people (11, 16, 50, 52). The finding that willingness to use PrEP was considerably lower when prescription requirements were involved further highlights access-related barriers that are well documented (16, 19, 53). These structural barriers are not unique to this setting, across sub-Saharan Africa, decentralisation of PrEP services, removal of prescription requirements, and integration of PrEP into community-based platforms have been identified as critical strategies for bridging the gap between intention and actual use (54, 55). Jointly, these findings reinforce the urgent need to address financial, structural, and informational barriers as interconnected determinants of PrEP uptake, rather than treating them as isolated programmatic challenges.

Years of study and age emerged as significant sociodemographic determinants of both PrEP knowledge and attitudes, with fourth year and older students demonstrating comparatively stronger performance across both domains. This pattern is consistent with evidence that cumulative academic exposure and professional socialisation within health science training progressively shape students’ health behaviour beliefs and engagement with biomedical prevention strategies (28, 30, 48). The influence of age further suggests that beyond formal education, broader life experience and psychosocial maturation may independently contribute to PrEP literacy and attitudinal receptivity. Notably, behavioural intention towards PrEP was not significantly predicted by any sociodemographic characteristic, suggesting that intention transcends individual demographics and is instead more powerfully shaped by structural factors such as cost, accessibility, and availability of information. This is consistent with broader evidence from sub-Saharan Africa indicating that structural barriers represent the primary determinants of PrEP uptake intention among young people (12, 14, 15). This underscores the importance of universally targeted campus-based interventions that prioritise the removal of shared structural barriers across the entire student population.

### Recommendations

Based on the findings of this study, several recommendations are proposed across different stakeholder levels. Students are encouraged to seek accurate PrEP information through campus student health and wellness centres and verified digital platforms. To critically evaluate social media health content and engage proactively with HIV prevention services including PrEP counselling offered through Higher Health-supported campus facilities. Higher education institutions should integrate comprehensive PrEP education, covering dosing, eligibility, mechanism of action, risk compensation concerns, and emerging long-acting formulations into health science curricula from the first year of study. Campus student health and wellness centres should be strengthened as accessible, stigma-free platforms for PrEP counselling and linkage to care, in collaboration with the Higher Education and Training HIV/AIDS Programme. The Department of Health should ensure sustained PrEP commodity supply to campus student health and wellness centres, expand access to long-acting injectable PrEP for young people, and support training of campus-based healthcare providers in PrEP counselling and prescription. Policymakers should prioritise the inclusion of campus-based PrEP programmes within the National Strategic Plan on HIV, TB and STIs, recognising higher education settings as strategic environments for HIV prevention. Finally, future research should assess health science students’ awareness, acceptability, and willingness to recommend long-acting injectable PrEP, while longitudinal and multi-institutional studies across Limpopo Province and broader sub-Saharan Africa are recommended to examine whether improved knowledge and positive attitudes translate into sustained PrEP uptake among healthcare graduates.

### Limitations

Several limitations of this study should be acknowledged. First, the cross-sectional design precludes causal inferences between sociodemographic characteristics and PrEP-related knowledge, attitudes, and behavioural intention; therefore, only associations can be interpreted. Second, the study was conducted at a single higher education institution in Limpopo Province, which may limit the generalisability of the findings to other institutional settings, provinces, and population groups. Third, the attitude scale demonstrated limited internal consistency (Cronbach’s *α* = 0.451), which may have affected the reliability and interpretability of the attitude-related findings. In addition, no adjustment for multiple comparisons was performed across the chi-square analyses, which may have increased the likelihood of Type I error and false-positive associations. Furthermore, behavioural intention was assessed as a proxy for actual PrEP uptake; therefore, longitudinal studies are warranted to determine whether stated intentions translate into actual utilisation of PrEP services. Finally, the exclusion of students from other health science disciplines, including Optometry and Human Nutrition and Dietetics, may have limited the comprehensiveness of the findings across the broader health science student population.

## Conclusion

This study demonstrated that the majority of health science students in Limpopo Province possessed good knowledge and positive attitudes towards HIV PrEP, with high behavioural intention to use PrEP as an HIV prevention strategy. These findings that are encouraging within the broader context of reducing advanced HIV disease burden in Sub-Saharan Africa. However, persistent knowledge gaps around dosing frequency and eligibility, alongside risk compensation concerns, highlight the urgent need for targeted, curriculum-integrated PrEP education within health science training programmes. The significant associations between year of study, age, and both knowledge and attitudes suggest that early integration of PrEP content into first-year curricula could accelerate PrEP literacy among future healthcare professionals. Existing campus-based health infrastructure supported by Higher Health presents a strategic opportunity to strengthen PrEP counselling, reduce stigma, and link students to available services, aligned with South Africa’s National Strategic Plan on HIV, TB and STIs. Furthermore, as frontline providers, Nursing and Pharmacy graduates represent a strategic investment in the prevention of advanced HIV disease and the achievement of the UNAIDS 95-95-95 targets in resource-limited settings.

## Data Availability

The original contributions presented in the study are included in the article/Supplementary material, further inquiries can be directed to the corresponding author.

## Supplementary material

Supplementary Figure S1 Sampling Flow Diagram. Flow diagram illustrating the target population, sample size determination using Slovin’s formula, participant recruitment process, response rates, and the final sample of nursing and pharmacy students included in the study.
