# Supplementary material for: Knowledge, attitudes, and behavioural intention towards HIV pre-exposure prophylaxis among health science students in South Africa: cross-sectional study
Source: Front Public Health. 2026 Jun 11;14:1858282. doi: 10.3389/fpubh.2026.1858282 (PMC13294217; doi:10.3389/fpubh.2026.1858282)
Supplement: Supplementary Figure S1 — Sampling Flow Diagram. Flow diagram illustrating the target population, sample size determination using Slovin’s formula, participant recruitment process, response rates, and the final sample of nursing and pharmacy students included in the study. [file Supplementary_file_1.DOCX]

Supplementary Material

**Supplementary Figure S1: Sampling Flow Diagram**
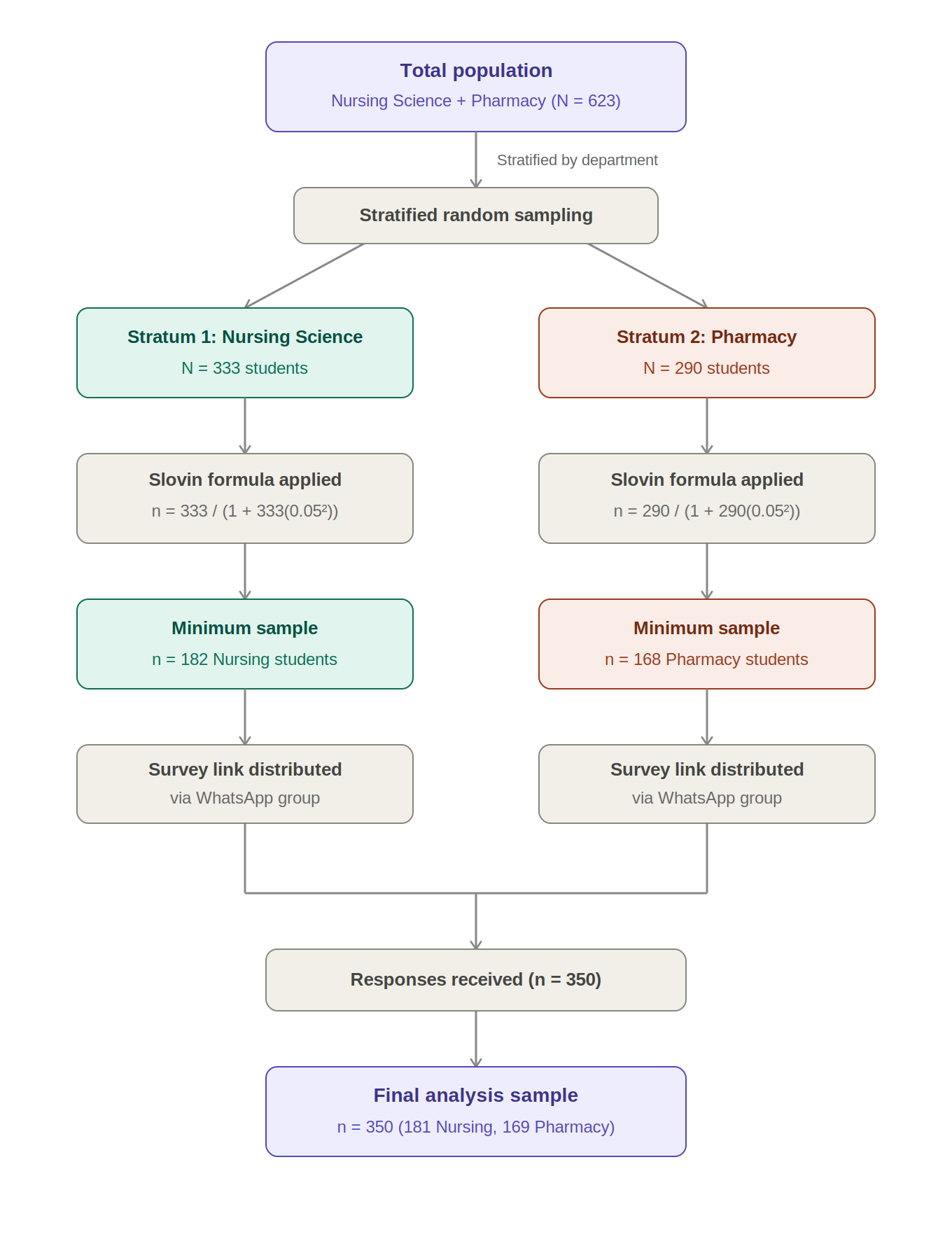


**Note.** *The minimum required sample size for each stratum was calculated separately using the Slovin formula: n = N / (1 + N(e²)), where n is the required sample size, N is the departmental population size, and e is the margin of error (0.05) at a 95% confidence level. This yielded a minimum required sample of 182 students from Nursing Science (N = 333) and 168 students from Pharmacy (N = 290), totalling a combined minimum required sample of 350 participants. A total of 350 valid responses were received and retained for the final analysis.*

**Supplementary Table S2:** Distribution of Responses to PrEP Knowledge Items (n = 350)

| Item | Response option | n | % |
| --- | --- | --- | --- |
| Have you ever heard of PrEP before? | Yes | 315 | 90.0 |
|  | No | 35 | 10.0 |
| What is PrEP? | Medication to prevent HIV | 307 | 87.7 |
|  | Vaccine against HIV | 23 | 6.6 |
|  | Treatment for HIV | 4 | 1.1 |
|  | I don’t know | 16 | 4.6 |
| How does PrEP work? | Prevents HIV from entering the body | 250 | 71.4 |
|  | Boosts the immune system | 43 | 12.3 |
|  | Kills HIV in the body | 12 | 3.4 |
|  | I don’t know | 45 | 12.9 |
| Who is eligible to take PrEP? | Anyone can take PrEP | 166 | 47.4 |
|  | Only people at high risk of HIV can take PrEP | 159 | 45.4 |
|  | I don’t know | 25 | 7.1 |
| How often should PrEP be taken? | Daily | 239 | 68.3 |
|  | Weekly | 6 | 1.7 |
|  | Monthly | 12 | 3.4 |
|  | I don’t know | 93 | 26.6 |
| What are the benefits of taking PrEP? | Reduces risk of HIV infection | 311 | 88.9 |
|  | Boosts immune system | 17 | 4.9 |
|  | Treats HIV infection | 2 | 0.6 |
|  | I don’t know | 20 | 5.7 |

**Supplementary Table S3:** Attitude Towards PrEP (Item-Level Responses)

| Item | Response Category | Frequency (n) | Percentage (%) |
| --- | --- | --- | --- |
| PrEP is an effective way to protect oneself from HIV | Yes | 269 | 76.9 |
|  | No | 29 | 8.3 |
|  | Maybe | 52 | 14.9 |
| PrEP promotes risky sexual behaviour | Yes | 170 | 48.6 |
|  | No | 113 | 32.3 |
|  | Maybe | 67 | 19.1 |
| Would consider taking PrEP if at high risk | Yes | 320 | 91.4 |
|  | No | 9 | 2.6 |
|  | Maybe | 21 | 6.0 |
| PrEP is an important HIV prevention strategy | Strongly agree | 172 | 49.1 |
|  | Agree | 129 | 36.9 |
|  | Neutral | 45 | 12.9 |
|  | Disagree | 2 | 0.6 |
|  | Strongly disagree | 2 | 0.6 |
| It is necessary to follow PrEP instructions strictly | Strongly agree | 254 | 72.6 |
|  | Agree | 78 | 22.3 |
|  | Uncertain | 16 | 4.6 |
|  | Disagree | 2 | 0.6 |
| Multiple sexual partners are acceptable when using PrEP | Strongly agree | 17 | 4.9 |
|  | Agree | 18 | 5.1 |
|  | Uncertain | 58 | 16.6 |
|  | Disagree | 110 | 31.4 |
|  | Strongly disagree | 147 | 42.0 |
| No need to use condoms when taking PrEP | Strongly agree | 12 | 3.4 |
|  | Agree | 10 | 2.9 |
|  | Uncertain | 33 | 9.4 |
|  | Disagree | 88 | 25.1 |
|  | Strongly disagree | 207 | 59.1 |

**Supplementary Table S4:** Behavioural Intention (Practice) Towards PrEP

| Item | Response Category | Frequency (n) | Percentage (%) |
| --- | --- | --- | --- |
| Would use PrEP as HIV prevention method | Yes | 259 | 74.0 |
|  | No | 38 | 10.9 |
|  | Maybe | 53 | 15.1 |
| Would be more willing if had more information | Yes | 276 | 78.9 |
|  | No | 19 | 5.4 |
|  | Maybe | 55 | 15.7 |
| Would be more willing if PrEP were free | Yes | 277 | 79.1 |
|  | No | 17 | 4.9 |
|  | Maybe | 56 | 16.0 |
| Would be more willing if available without prescription | Yes | 178 | 50.9 |
|  | No | 115 | 32.9 |
|  | Maybe | 57 | 16.3 |

**Notes:** *Participant characteristics (n = 350); n = number of participants. SD = Standard Deviation. Percentages may not sum to 100% due to rounding.*
